# Is photobiomodulation therapy free from racial bias?: a narrative review of skin pigmentation

**DOI:** 10.1590/1516-3180.2025.3358.26012026

**Published:** 2026-04-10

**Authors:** Carlos Eduardo Girasol, Luciano Bachmann

**Affiliations:** I Postdoctoral Fellow, Researcher, and Clinical Physiotherapist; Departamento de Física, Faculdade de Filosofia, Ciências e Letras de Ribeirão Preto, Universidade de São Paulo (FFCLRP-USP), Ribeirão Preto (SP), Brazil.; II Professor, Researcher; Departamento de Física, Faculdade de Filosofia, Ciências e Letras de Ribeirão Preto, Universidade de São Paulo (FFCLRP-USP), Ribeirão Preto (SP), Brazil.

**Keywords:** Melanins, Laser therapy, Skin, Light-emitting diode, Low-level light therapy, Melanin, Phototherapy, Physical therapy, Skin

## Abstract

**BACKGROUND:**

Photobiomodulation therapy (PBMT) has been extensively researched for tissue repair, pain relief, and muscle recovery. However, melanin, a primary skin chromophore, can impede photon penetration into darker skin, potentially diminishing the efficacy of PBMT. Despite this, clinical protocols and guidelines seldom account for skin pigmentation when setting parameters, possibly exacerbating racial disparities in healthcare.

**OBJECTIVE:**

To examine the effect of melanin on the efficacy of PBMT and highlight the necessity for personalized strategies that account for skin pigmentation.

**DESIGN AND SETTING:**

Short communication conducted at the Universidade de São Paulo (USP), Ribeirão Preto, São Paulo.

**METHODS:**

A narrative review of PBMT mechanisms, light–tissue interactions, and the impact of melanin absorption on treatment outcomes was conducted.

**RESULTS:**

The clinical outcomes of PBMT depend on the technical parameters (wavelength, energy, and dose) and skin pigmentation. Darker skin tones with higher melanin content may lead to reduced photon penetration effectiveness. However, most studies and protocols do not consider this variable. Only a few clinical trials have categorized outcomes by skin tone, exposing a notable knowledge gap.

**CONCLUSION:**

Melanin plays a crucial role in PBMT response, and overlooking skin pigmentation in research and clinical practice could perpetuate disparities. Enhanced and tailored protocols are essential to optimize PBMT outcomes among diverse populations.

## INTRODUCTION

 Photobiomodulation therapy (PBMT) has been widely studied for its effects on tissue repair, analgesia, and muscle recovery;^1^ however, its mechanisms are only partially understood. The process involves the absorption of photons by mitochondrial chromophores, which leads to ATP synthesis, the modulation of reactive species, and subsequent signaling pathways.^2^ The therapeutic effectiveness of PBMT is affected by various physical and biological factors. Parameters such as wavelength, power, and energy play a critical role in determining the interaction of light with tissues. The therapeutic outcome is dependent on both technical (wavelength, power, and energy) and biological factors. In the “optical window” (600–1100 nm), light transmission is optimized, although chromophores such as melanin, hemoglobin, and water significantly affect the propagation of photons.^3,4^

 Melanin is particularly significant due to its wide absorption spectrum, potentially restricting light penetration in darker skin tones. Optical studies^5,6^ have shown that device characteristics and tissue properties can unpredictably affect penetration, raising concerns about standardization. Despite this, prominent guidelines such as those from the World Association for Photobiomodulation Therapy (2010, 2022) currently do not differentiate dosing parameters based on skin pigmentation. In regions with predominantly Black or Brown populations, this oversight may worsen inequities. Furthermore, limited clinical studies have categorized participants by skin type, resulting in the underrepresentation of melanodermic individuals in the literature. 

## IS MELANIN THE MAIN CULPRIT?

 In PBMT, chromophores absorb light to initiate biological responses. Key endogenous chromophores found in human tissues comprise melanin, hemoglobin, water, and cytochrome c-oxidase.^2^ Melanin is particularly noteworthy due to its robust and broad-spectrum absorption, particularly across the visible and near-infrared spectra (600–900 nm) commonly employed in PBMT. While melanin plays a crucial role in protecting the skin from photodamage, it also acts as a substantial optical impediment.^7^ In individuals with darker skin tones, heightened melanin levels can absorb a significant portion of incoming photons, thereby diminishing the energy penetrating deeper tissues and potentially reducing the therapeutic efficacy.^3^

 Importantly, melanin concentration and distribution vary among individuals and across anatomical regions. However, these factors are rarely considered in standard PBMT protocols. This oversight can lead to mismatches between the planned and delivered energy, resulting in suboptimal or inconsistent outcomes, particularly in melanodermic populations. Optical studies have confirmed that melanin significantly affects light distribution and penetration depth.^7^

 The wide absorption spectrum of melanin enables it to absorb a significant amount of incident light, reducing the availability of photons for therapeutic chromophores. However, its role extends beyond simple attenuation of incident fluence. Upon excitation, melanin can trigger photochemical reactions that generate heat and multiple reactive oxygen and nitrogen species (ROS/RNS), including singlet oxygen, hydrogen peroxide, superoxide, hydroxyl radicals, and nitric oxide derivatives. These pathways reflect the dual photobiology of melanin: protective in certain circumstances but reactive and potentially harmful in others.^2,8^ This dual nature is particularly pertinent when adjusting parameters to enhance the penetration of PBMT into darker skin. While increasing the fluence, irradiance, or exposure time can offset superficial absorption, these adjustments also raise the amount of energy deposited in the melanin-rich epidermis. This elevation escalates the risk of thermal burden, localized heat buildup, and heightened ROS/RNS production, which may clinically present as discomfort, erythema, or superficial injury.^8^

 The issue at hand is not whether to increase PBMT parameters, but how to execute them safely. Evidence from optical modeling and clinical studies advocates for several strategies: giving preference to longer wavelengths (e.g., 800–900 nm and 1.064 nm), which exhibit reduced melanin absorption; utilizing larger spot sizes to lessen epidermal burden and enhance depth penetration; monitoring initial sensory cues such as warmth or tingling, which manifest at lower fluences in darker phototypes; and implementing gradual rather than sudden fluence increments. In summary, melanin does not impede PBMT but serves as a crucial dosimetric factor. Incorporating its optical behavior into treatment planning is essential for maximizing efficacy while maintaining safety. 

## LIGHT IS LIGHT? RETHINKING STANDARDIZATION IN OPTICAL THERAPIES

 Despite advances in PBMT, uncertainties persist when these protocols are applied to diverse populations. Optical technologies encounter inherent limitations, such as device variability, disparities between programmed and delivered parameters, and the biological diversity of human skin. Factors like melanin content, tissue thickness, and individual optical responses are frequently overlooked in clinical practice and research. 

 A systematic review of 461 trials on cosmetic laser- and light-based therapies identified structural bias.^9^ While many trials included phototypes IV–VI, few analyzed outcomes based on skin type, compromising their relevance to darker-skinned populations. Conditions like post-inflammatory hyperpigmentation frequently preclude higher phototypes from primary laser treatments due to variable efficacy and a higher risk of adverse effects. Despite the promise of techniques such as fractional photothermolysis and Nd:YAG when used judiciously, the literature is still fragmented and heavily dependent on subjective skin classification scales.^10^

 Taken together, these gaps emphasize the need to reassess whether the current PBMT approaches sufficiently account for individual variability. As Enwemeka^11^ noted, "light is light," but biological diversity requires moving beyond technical precision to clinically inclusive applications. 

## WHAT SHOULD BE CONSIDERED WITH CAUTION?

 Patient responses to PBMT vary depending on pigmentation, tissue composition, and perception thresholds. Individuals with darker phototypes may experience warmth or discomfort at lower fluences. Sartor et al.^12^ demonstrated that individuals with higher phototypes perceived PBMT stimuli sooner, even with standardized parameters, highlighting the importance of carefully adjusting energy density and duration to prevent adverse effects. Tattoos and other pigment alterations can change cutaneous perception, complicating treatment planning. 

 Experimental models^4^ and in vivo optical studies^6^ further confirmed the reduced penetration at 660 nm in darker skin compared with lighter tones, with wavelength-dependent differences (e.g., 14 versus 21 mm for 660 nm; 20 versus 26 mm for 830 nm). Equipment variability adds further inconsistencies. These findings challenge one-size-fits-all protocols; clinicians must assess the pigmentation, anatomical site, and tissue thickness to adapt the parameters. Insights from dermatological laser therapy suggest that PBMT should adopt a pigmentation-based calibration to enhance its safety and efficacy. This naturally leads to the challenge of individualized dosimetry in PBMT. How can we accurately measure and adjust the treatment parameters to consider variations in melanin concentration, tissue optical properties, and other patient-specific factors? Addressing this issue is crucial for advancing photobiomodulation into a personalized therapeutic modality. 

## WHERE DO WE GO FROM HERE? A CALL FOR INDIVIDUALIZED DOSIMETRY

 The notion that PBMT is universally effective regardless of skin tone is oversimplified. Individuals with higher melanin concentrations may receive subtherapeutic fluences if protocols are not adjusted, leading to concerns about effectiveness and fairness. Future studies should adopt individualized dosimetry, consider skin phototype as a key factor, and involve participants from all Fitzpatrick categories. Computational models can aid in refining treatment strategies. Clinicians should adjust the wavelength, power, and exposure according to individual patient characteristics. The integration of pigmentation-sensitive dosimetry is crucial for the progress of PBMT in personalized treatment. 

## CONCLUSION

 Melanin is a crucial factor influencing the results of PBMT. Standardized protocols that ignore the risk of skin pigmentation lead to disparities in treatment efficacy and safety. Integrating pigmentation assessment and individualized dosimetry into research and clinical practice is essential for advancing PBMT into a more effective, safe, and inclusive therapeutic modality. 

## CLINICAL RELEVANCE

- PBMT outcomes vary with skin pigmentation, emphasizing the necessity for individualized dosimetry.
- Adjustment of parameters for melanin improved safety and efficacy.
- Pigmentation-aware protocols ensure consistent, equitable treatment.

## Data Availability

Data supporting the findings of this study are available from the corresponding author, Carlos Eduardo Girasol, upon request.
